# The Effective Management of Fever in Pediatrics and Insights on Remote Management: Experts' Consensus Using a Delphi Approach

**DOI:** 10.3389/fped.2022.834673

**Published:** 2022-04-26

**Authors:** Elena Chiappini, Antonio Vitale, Raffaele Badolato, Paolo Becherucci, Domenico Careddu, Antonio Di Mauro, Mattia Doria, Annamaria Staiano

**Affiliations:** ^1^Pediatric Infectious Disease Unit, Anna Meyer Children's University Hospital, Florence, Italy; ^2^Department of Pediatrics, Azienda Ospedaliera San Giuseppe Moscati, Avellino, Italy; ^3^Department of Clinical and Experimental Sciences, Pediatrics Clinic, University of Brescia and ASST-Spedali Civili, Brescia, Italy; ^4^Pediatric Primary Care, National Pediatric Health Care System, Lastra a Signa, Italy; ^5^Pediatric Primary Care, National Pediatric Health Care System, Novara, Italy; ^6^Pediatric Primary Care, National Pediatric Health Care System, Margherita di Savoia, Barletta-Andria-Trani, Italy; ^7^Pediatric Primary Care, National Pediatric Health Care System, Chioggia, Italy; ^8^Department of Translational Medical Science, Section of Pediatrics, University of Naples Federico II, Naples, Italy

**Keywords:** fever, pediatrics, Delphi process, remote management, expert consensus

## Abstract

**Background:**

Even after the publication of the 2017 update of Italian guidelines on treatment of fever in pediatrics, some fundamental questions are still open and new ones emerged during the COVID-19 pandemic.

**Objective:**

To assess the level of consensus among Italian pediatricians on different topics related to treatment of fever in children by using the Delphi technique.

**Methods:**

A Delphi study was undertaken between June and September 2021, when two questionnaires were consecutively sent to a panel of experts to be answered anonymously. An invitation to participate was sent to 500 pediatricians distributed over the whole national territory and 80 (16%) of them accepted to participate on a voluntary basis. The questionnaires were structured into three specific topics: “therapeutic appropriateness and management of the febrile child,” “management of the febrile child in the presence of other diseases,” and “future perspectives in remote management.” Each topic had six statements.

**Results:**

A first-round questionnaire was sent to 80 accepting pediatricians from different Italian regions. Of the 72 respondents (23% working in hospitals and 72% outside), 33% were from northern, 12% central, and 55% southern Italy or islands. A second-round questionnaire was sent to the same 80 pediatricians and 69 of them responded, without significant differences for workplaces or geographical distribution as compared with the first questionnaire. Overall, 75 participants answered at least one of the two questionnaires. All the statements on the topics of “therapeutic appropriateness and management of the febrile child” and “future perspectives in remote management” reached the predefined cut off for consensus (75% or more). Only one statement on “management of the febrile child in the presence of other diseases” did not achieve the consensus even after the second round.

**Conclusions:**

Italian pediatricians agree on several aspects of treatment of febrile children and their expert opinions could support everyday decision process complementary to recommendations by regulatory agencies and guidelines.

## Introduction

In 2017, the Italian Society of Pediatrics published an update of the guidelines on the management of fever in children (1). However, differences may have persisted among regions and between settings, namely, hospital vs. community, and may have further increased with the outbreak of SARS-CoV-2 virus pandemic (COVID-19), due to limited resource availability and social containment measures. Moreover, the pandemic has stressed the difficulties for local services in securing the continuity of care to patients suffering from rare or common diseases, difficulties that a remote approach could have reduced. Therefore, the COVID-19 outbreak has raised questions that weaken the applicability of the available guidelines to everyday clinical practice.

In making clinical decisions where no gold standard exists or when new situations, like COVID-19, emerge, formal consensus techniques among peers represent a recognized method of scientific advancement based on clinical experience and an authoritative opinion to support therapeutic decisions in clinical practice (2). One of the most validated formal methods for consensus processes in various fields is the Delphi method (3). It consists of an iterative process in which experts are consulted individually with a series of questionnaires and receive anonymous group feedback from authoritative experts between iterations.

In this study, we used a modified Delphi process to assess the level of consensus among pediatricians from different Italian regions and workplaces on the management of pediatric fever, in terms of both therapeutic appropriateness and management in the presence of other diseases, including COVID-19 infection. The possible role of remote management of the patient was also evaluated, with reference to both the current situation and other similar possible future situations. The ultimate output was to propose statements made by a board of pediatric experts about a standard treatment to be applied in daily practice for the febrile child, which could also be feasible with a remote approach. The study does not aim to provide guidelines.

## Methods

**Figure 1:**
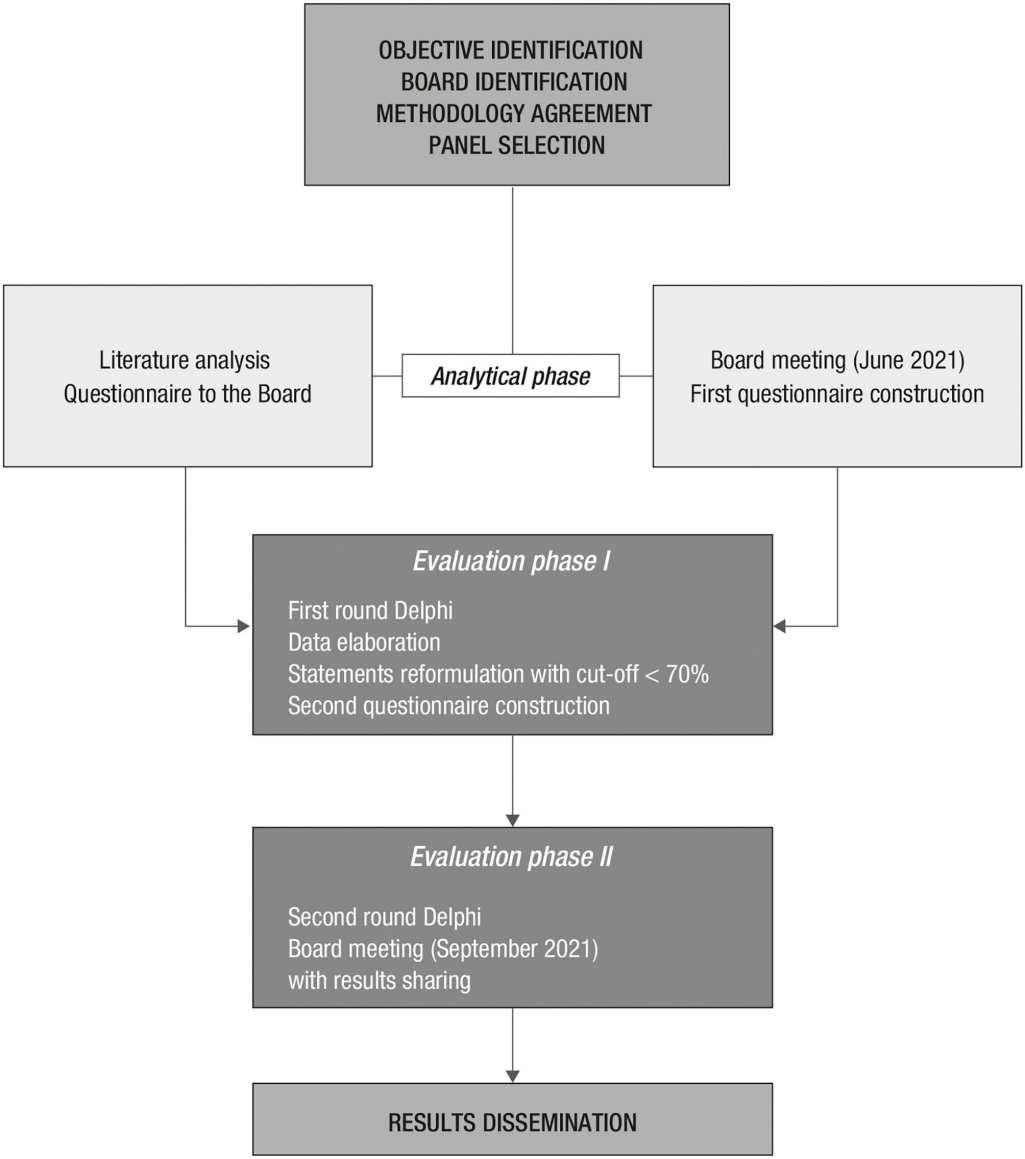
Delphi method flow chart.

The study was conducted between June and September 2021 (Figure 1) among Italian pediatricians with relevant clinical experience in the treatment of the febrile patient, operating in either hospital or community settings (see Appendix).

### Exploratory Phase

A board of pediatricians working in academic institutions or hospitals or community settings with ≥10 years of clinical experience, active participation in boards and committees of the Italian Society of Pediatrics and other scientific societies, and at least 10 international publications on the topic of fever management was identified (the authors of the current study).

A panel of board-certified pediatricians was selected from a database of Menthalia S.r.l., a provider for scientific and medical communication. They were required to have the following qualifications: clinical experience in the pediatric field for 5 years at least, working in hospitals or primary care settings over the Italian territory, at least 5 scientific publications on fever treatment in the last 5 years, and participation in national or international meetings and in the activities of scientific societies. A total of 500 pediatricians distributed over the country were invited by Menthalia and 80 (16%) accepted to participate on a voluntary basis, which was considered as acceptable.

### Analytical Phase

Guidelines and consensus documents on pediatric fever management were initially collected to assess the state of the art at the national level. A literature search was performed in May 2021 on PubMed by using the PICO criteria:

- Population: children (age: 0–18 years).
- Intervention: medical management.
- Comparators: paracetamol vs. ibuprofen.
- Outcomes: subjective and objective.
- Areas: outpatient and inpatient.

The search included randomized clinical trials, observational studies (prospective or retrospective cohorts, case-control or cross-sectional studies), systematic reviews and meta-analyses, and guidelines published between 2015 and 2021. Publications in languages other than English or Italian, case reports, letters, editorials, and gray literature were not included.

The Boolean terms used for the search were:

- children AND fever management NOT cancer (344 results)
- children AND fever management AND therapeutic appropriateness (27 results)
- children AND fever management AND paracetamol (22 results)
- fever management AND pediatric patients AND paracetamol NOT cancer (2 results)
- children AND fever management AND AOM (acute otitis media) (6 results)
- children AND fever management AND asthma (7 results)
- children AND fever management AND kidney disease (5 results)
- children AND fever management AND sinusitis (2 results)
- children AND fever management AND COVID-19 (29 results)
- children AND fever management AND telemedicine (3 results).

After a preliminary analysis of the titles and abstracts from all search results, the appropriateness of the contents with respect to the objective of the study, the geographical area of reference, and the treated diseases were evaluated. Duplicates, papers on critically ill children, or fever management in developing countries were discarded.

Finally, 29 papers relevant to the objective of the study were retained, to which three relevant documents related to telemedicine were added, and divided into the following four areas of interest:

1. Therapeutic appropriateness and management of the febrile child.
2. Management of the febrile child in the presence of other diseases.
3. Management of fever and COVID-19.
4. Future perspectives: remote management.

### Delphi Process

Based on the literature search (1, 4–18), a questionnaire (Table 1) was prepared by one of the board members (Antonio Vitale) and submitted to the board, in a non-anonymous manner, in order to identify areas of agreement and acquire further information to improve the survey through open questions. The responses of the board members to each statement were analyzed by a descriptive statistic performed by Menthalia and discussed during two web meeting brainstorms, moderated by a facilitator from Menthalia. The discussion led to reformulating some statements and deleting others. The section on COVID-19 was omitted, and only one statement on it was included in the section on febrile child with other diseases.

**Table 1:** Questionnaire administered to the board.

**Therapeutic appropriateness and management of the febrile child**	
**1**	Paracetamol is the only antipyretic and pain reliever that can be used from birth to 3 months of age.
**2**	Paracetamol and ibuprofen are the only highly recommended antipyretics in children.
**3**	In the treatment of febrile children, monotherapy is recommended as opposed to the combined or alternating use of paracetamol and ibuprofen.
**4**	In febrile and/or painful children the optimal dosage of ibuprofen should not exceed 10 mg/kg 3 times a day.
**5**	In febrile and/or painful children the optimal dosage of paracetamol should not exceed 15 mg/kg 4 times a day.
**6**	Given its effectiveness as an antipyretic and greater tolerability, paracetamol, at recommended doses, is the drug to be used as the first choice in febrile children.
**Management of the febrile child in the presence of other diseases**	
**1**	In the presence of acute otitis media (AOM), ibuprofen, at the recommended doses, is more appropriate than paracetamol.
**2**	In the presence of asthma, paracetamol, at recommended doses, is more appropriate than ibuprofen.
**3**	In the presence of dehydration or gastrointestinal problems, paracetamol, at the recommended doses, is more appropriate than ibuprofen.
**4**	In the presence of severe liver disease, paracetamol, at the recommended doses, is more appropriate than ibuprofen.
**Management of fever and COVID-19**	
**1**	In children with COVID-19, monotherapy with paracetamol as the first-choice antipyretic is recommended.
**2**	In SARS-CoV-2 infections with a benign course requiring only supportive therapy, paracetamol is preferred over ibuprofen.
**3**	Appropriate therapeutic management combined with monitoring for the next 4 weeks of the pediatric patient with SARS-CoV-2 could avoid the onset of a multisystem inflammatory syndrome (MIS-C).
**4**	In a pandemic or post-pandemic period, telemedicine and remote monitoring (WhatsApp, Skype, SMS, etc.) should be used with strict hospital admission criteria.
**Future perspectives: remote management**	
**1**	The models of care that emerged in the pandemic period with remote management and strict hospital admission criteria can become, with the appropriate adaptations, a model to be followed also in the future.
**2**	The management of fever and pain should be organized with an integrated approach that includes the assessment in the presence and remote monitoring of a set of indicators (color, body temperature, absence of reaction to stimuli, headache, etc.).
**3**	Telemedicine and more generally the remote management of pediatric diseases can increase access to care.
**4**	The use of platforms, Apps and messaging systems (WhatsApp, Skype, SMS, etc.) for monitoring the disease in pediatric patients is desirable.
**5**	It is desirable to develop training programs for the use of platforms, Apps and messaging systems in the management of the pediatric patient.

A first questionnaire for anonymous consultation with the panel was then formulated, consisting of 18 statements divided into three sections (Table 2). Two questions related to panelists' geographical areas (north, center, or south) and workplace (hospital or territorial) were added. After the first round of consultation, the board processed the answers given by the panelists and produced a second questionnaire, which was eventually submitted to the same panel of experts.

**Table 2:** First questionnaire administered to the panel.

**Therapeutic appropriateness and management of the febrile child**	
**1**	The goal of paracetamol therapy is to reduce malaise in a febrile child.
**2**	Paracetamol and ibuprofen are the only highly recommended antipyretics in children.
**3**	Paracetamol is the only antipyretic and pain reliever that can be used from birth to 3 months of age with a recommended dose of 10 mg/kg orally every 6 h.
**4**	In febrile and painful children over three months of age, the recommended dose of ibuprofen is 20–30 mg/kg/day orally (in 3 doses every 8 h).
**5**	In febrile and painful children over 3 months of age, the recommended dose of paracetamol is 15 mg/kg every 6 h.
**6**	Given its effectiveness as an antipyretic and greater tolerability, paracetamol, at recommended doses, is the drug to be used as the first choice in febrile children.
**Management of the febrile child in the presence of other diseases**	
**1**	In the presence of acute otitis media (AOM), ibuprofen at the recommended doses is more effective to relieve pain than paracetamol.
**2**	In the presence of asthma, paracetamol, at recommended doses, is more appropriate than ibuprofen.
**3**	In the presence of dehydration, with or without acute gastroenteritis, paracetamol, at the recommended doses, is the only drug to be recommended.
**4**	In case of suspicion of lower respiratory tract infections (LRTI) to reduce the risk of developing pneumonia, administration of paracetamol, at recommended doses, is more appropriate than ibuprofen.
**5**	In the presence of severe renal disease, the administration of paracetamol, at the recommended doses, is more appropriate than ibuprofen.
**6**	In the presence of SARS-CoV-2 infection with a benign course requiring only supportive therapy, paracetamol monotherapy, in the presence of fever > 38°C, is preferred.
**Future perspectives: remote management**	
**1**	In a pandemic or post-pandemic period, telemedicine and clinical and instrumental monitoring can be used remotely and following the guidelines appropriate for each clinical condition.
**2**	Telemedicine and more generally the remote management of pediatric diseases can increase access to care.
**3**	Telemedicine should be considered as a tool for integrating direct clinical practice and/or used as a follow-up activity.
**4**	It is desirable to develop digital tools which, by monitoring clinical and instrumental indicators, warn in real time of the appearance of alarm signs and symptoms.
**5**	It is desirable to develop an App for relatives and caregivers from an authoritative and certified source to improve adherence to therapy.
**6**	It is desirable to develop training programs for the use of new-generation digital tools aimed at clinicians, parents and caregivers.

### Evaluation Phase

The questionnaires shared by the board were administered to the panel of experts *via* a web platform that guaranteed anonymity and the impossibility for the manager to associate the single questionnaire with the compiler. Each panelist had previously received the literature references supporting the questionnaire. Panelists were asked to score each statement by the following scale: 1, strong disagreement; 2, fair disagreement; 3, no opinion; 4, fair agreement; 5, strong agreement. For the analysis of results, responses were categorized as negative (score 1–2) or neutral (score 3), and positive (score 4–5). For each statement, the average score and the percentage of voters who gave positive responses were considered, and the cut-off level for consensus was 75% agreement. After the first round, the board evaluated the responses in order to identify areas of agreement and acquire further information to improve the survey through a second questionnaire (Table 3).

**Table 3:** Second questionnaire administered to the panel.

**Therapeutic appropriateness and management of the febrile child**	
**1**	The goal of paracetamol therapy is to reduce malaise in a febrile child.
**2**	Paracetamol and ibuprofen are the only highly recommended antipyretics in children.
**3**	Paracetamol is the only antipyretic and pain reliever that can be used from birth to 3 months of age with a recommended dose of 10 mg/kg orally every 6 h.
**4**	In febrile and painful children over 3 months of age, the recommended dose of ibuprofen is 20–30 mg/kg/day orally (in 3 doses every 8 h).
**5**	In febrile and painful children over 3 months of age, the recommended dose of paracetamol is 15 mg/kg every 6 h.
**6**	Given its effectiveness as an antipyretic and greater tolerability, paracetamol, at recommended doses, is the drug to be used as the first choice in febrile children.
**Management of the febrile child in the presence of other diseases**	
**1**	In the presence of acute otitis media (AOM), ibuprofen and paracetamol, at the recommended doses, are equivalent.
**2**	In the presence of asthma, paracetamol, at recommended doses, is more appropriate than ibuprofen.
**3**	In the presence of dehydration, with or without acute gastroenteritis, paracetamol, at the recommended doses, is the only drug to be recommended.
**4**	In case of suspicion of lower respiratory tract infections (LRTI) the administration of paracetamol, at the recommended doses, is more appropriate than ibuprofen.
**5**	In the presence of severe renal disease, the administration of paracetamol, at the recommended doses, is more appropriate than ibuprofen.
**6**	In the presence of SARS-CoV-2 infection, monotherapy with paracetamol as the first-choice antipyretic is recommended.
**Future perspectives: remote management**	
**1**	In a pandemic or post-pandemic period, telemedicine and clinical and instrumental monitoring can be used remotely and following the guidelines appropriate for each clinical condition.
**2**	Telemedicine and more generally the remote management of pediatric diseases can increase access to care.
**3**	Telemedicine should be considered as a tool for integrating direct clinical practice and/or used as a follow-up activity.
**4**	It is desirable to develop digital tools which, by monitoring clinical and instrumental indicators, warn in real time of the appearance of alarm signs and symptoms.
**5**	It is desirable to develop an App for relatives and caregivers from an authoritative and certified source to improve adherence to therapy.
**6**	It is desirable to develop training programs for the use of new-generation digital tools aimed at clinicians, parents and caregivers.

## Results

The first questionnaire administered to the panel (Table 2) was sent to 80 pediatricians, and 72 of them responded. Of them, 23% were working in hospitals and 72% outside, 33% were from northern, 12% central, and 55% southern Italy or islands. The second questionnaire administered to the panel (Table 3) was sent to the same 80 pediatricians and 69 of them responded, without significant differences for workplaces or geographical distribution as compared with the first questionnaire. Overall, 75 participants answered at least one of the two questionnaires.

The results of both rounds are presented in Tables 4a–c. After the first round, there was a high level of consensus for all the statements of the first section, which is “Therapeutic appropriateness and management of the febrile child,” with average scores from 4.6 to 4.8 and over 90% of positive responses. For the second section, “Management of the febrile child in the presence of other diseases,” there was consensus level on three statements only, namely, on asthma, dehydration, and severe renal disease, with average scores from 4.2 to 4.3 and agreement from 79% to 84%. Three statements, namely, on AOM, lower respiratory tract infections, and COVID-19, obtained average scores from 3.4 to 4.0 and agreement from 55% to 70%, thus below the level of consensus. For the third section, “Future perspectives: remote management,” there was good consensus on all statements, with average scores from 4.0 to 4.6 and agreement from 77% to 96%.

**Table 4a:** Summary results of the two Delphi consultations.

		**First round**		**Second round**	
		**Mean score**	**Agreement** **on 4 and 5**	**Mean score**	**Agreement** **on 4 and 5**
	**Therapeutic appropriateness and management of the febrile child**				
**1**	The goal of paracetamol therapy is to reduce malaise in a febrile child.	4.8	97.0%	4.8	100%
**2**	Paracetamol and ibuprofen are the only antipyretics recommended in children.	4.8	98.5%	4.8	98.6%
**3**	Paracetamol is the only antipyretic and pain reliever that can be used from birth to 3 months of age with a recommended dose of 10 mg/kg orally every 6 h.	4.6	93.9%	4.7	98.6%
**4**	In febrile and painful children over 3 months of age, the recommended dose of ibuprofen is 20–30 mg/kg/day orally (in 3 doses every 8 h).	4.6	92.4%	4.6	95.7%
**5**	In febrile and painful children over 3 months of age, the recommended dose of paracetamol is 15 mg/kg every 6 h.	4.7	97.0%	4.7	97.1%
**6**	Given its efficacy as an antipyretic and greater tolerability, paracetamol, at recommended doses, is the drug to be used as the first choice in febrile children.	4.8	95.5%	4.8	97.1%

*Scoring scale: 1, strong disagreement; 2, fair disagreement; 3, no opinion; 4, fair agreement; 5, strong agreement*.

**Table 4b:** Summary results of the two Delphi consultations.

		**First round**		**Second round**	
		**Mean score**	**Agreement** **on 4 and 5**	**Mean score**	**Agreement** **on 4 and 5**
	**Management of the febrile child in the presence of other diseases**				
**2**	In the presence of asthma, paracetamol, at recommended doses, is more appropriate than ibuprofen.	4.2	78.8%	4.2	72.5%
**3**	In the presence of dehydration, with or without acute gastroenteritis, paracetamol, at the recommended doses, is the only drug to be recommended.	4.3	83.3%	4.3	82.6%
**5**	In the presence of severe renal disease, the administration of paracetamol, at the recommended doses, is more appropriate than ibuprofen.	4.3	81.8%	4.5	88.4%
**1**	In the presence of acute otitis media (AOM), ibuprofen at the recommended doses is more effective to relieve pain than paracetamol.	3.4	54.5%		
**4**	In case of suspicion of lower respiratory tract infections (LRTI) to reduce the risk of developing pneumonia, administration of paracetamol, at recommended doses, is more appropriate than ibuprofen.	3.8	66.7%		
**6**	In the presence of SARS-CoV-2 infection with a benign course requiring only supportive therapy, paracetamol monotherapy, in the presence of fever > 38°C, is preferred.	4.0	69.7%		
**1**	In the presence of acute otitis media (AOM), ibuprofen and paracetamol, at the recommended doses, are equivalent.			4.1	76.8%
**4**	In case of suspicion of lower respiratory tract infections (LRTI) the administration of paracetamol, at the recommended doses, is more appropriate than ibuprofen.			3.3	46.4%
**6**	In the presence of SARS-CoV-2 infection, monotherapy with paracetamol as the first-choice antipyretic is recommended.			4.4	84.1%

*Scoring scale: 1, strong disagreement; 2, fair disagreement; 3, no opinion; 4, fair agreement; 5, strong agreement*.

**Table 4c:** Summary results of the two Delphi consultations.

		**First round**		**Second round**	
		**Mean score**	**Agreement** **on 4 and 5**	**Mean score**	**Agreement** **on 4 and 5**
	**Future perspectives: remote management**				
**1**	In a pandemic or post-pandemic period, telemedicine and clinical and instrumental monitoring can be used remotely and following the guidelines appropriate for each clinical condition.	4.3	83.3%	4.4	92.8%
**2**	Telemedicine and more generally the remote management of pediatric diseases can increase access to care.	4.0	77.3%	4.2	81.2%
**3**	Telemedicine should be considered as a tool for integrating direct clinical practice and/or used as a follow-up activity.	4.4	93.9%	4.5	91.3%
**4**	It is desirable to develop digital tools which, by monitoring clinical and instrumental indicators, warn in real time of the appearance of alarm signs and symptoms.	4.5	90.9%	4.4	89.9%
**5**	It is desirable to develop an App for relatives and caregivers from an authoritative and certified source to improve adherence to therapy.	4.3	89.4%	4.5	89.9%
**6**	It is desirable to develop training programs for the use of new-generation digital tools aimed at clinicians, parents and caregivers.	4.6	95.5%	4.6	92.8%

*Scoring scale: 1, strong disagreement; 2, fair disagreement; 3, no opinion; 4, fair agreement; 5, strong agreement*.

Therefore, based on the results of the first round, the board modified statements for the second round. Statement no. 1, “In the presence of acute otitis media (AOM), ibuprofen at the recommended doses is more effective to relieve pain than paracetamol,” became “In the presence of acute otitis media (AOM), ibuprofen and paracetamol, at recommended doses are equivalent.” Statement no. 4, “In case of suspicion of lower respiratory tract infections (LRTI) to reduce the risk of developing pneumonia, the administration of paracetamol, at the recommended doses, is more appropriate than ibuprofen,” became “In case of suspicion of lower respiratory tract infections (LRTI), the administration of paracetamol, at the recommended doses, is more appropriate than ibuprofen.” Statement no. 6, “In the presence of SARS-CoV-2 infection with a benign course requiring only supportive therapy, monotherapy with paracetamol, in the presence of fever> 38°C, is preferred,” became “In the presence of SARS-CoV-2 infection, monotherapy with paracetamol as the first-choice antipyretic is recommended.”

The results of the second round confirmed the consensus on all previous statements and achieved the consensus on statements no. 1 and 6 as modified. Despite the modification, statement no. 4 did not achieve the required level of consensus.

## Discussion

The main finding of this study was that a panel of Italian pediatricians agreed on most of the statements on the management of febrile children.

### Therapeutic Appropriateness and Management of the Febrile Child

The first section of the questionnaire included statements mostly regarding the indications and dosage of paracetamol and ibuprofen in febrile children, as per regulatory and guidelines recommendations. The excellent levels of consensus achieved after both rounds suggest that the panelists of the present study consider, based on their clinical experience, the recommended use and dosage of these antipyretic drugs (paracetamol and ibuprofen) adequate even for very young children (10), which was not always the case in the past. The statement no. 6 on paracetamol as first-choice antipyretic in children achieved a high level of consensus. In the absence of an explicit recommendation by the current guidelines, this may reflect homogeneity of opinions derived from individual clinical practice.

### Management of the Febrile Child in the Presence of Other Diseases

The second section of the questionnaire included statements on issues less disciplined by guidelines or supported by a limited number of studies (11–17). As expected, the agreement was variable, with only three statements, which focused on potential contraindications rather than efficacy, achieving the consensus level already after first round. Of the three statements not achieving consensus after the first round, no. 1 was focused on pain rather than fever, no. 2 on a clinically not well-defined condition as a preventive treatment to reduce complications, and no. 3 on COVID-19 domiciliary treatment slightly differing from the current recommendations by the Italian government. The disagreement on statement no. 1 likely reflects that the panelists did not consider ibuprofen as having superior analgesic properties, which was confirmed by the consensus achieved at the end of the second round on the modified statement, where pain was omitted with paracetamol and ibuprofen being considered equivalent. Similarly, the consensus on statement no. 6 was achieved at the second round after the limitation to fever > 38°C had been removed. This is partially in line with the protocol of the Italian government for general COVID-19 population, which recommends the use of paracetamol or non-steroidal anti-inflammatory drugs without fever threshold.

### Future Perspectives: Remote Management

The third section of the questionnaire was focused on remote medical management, not specifically limited to febrile children (18). Although this is a topic not widely considered in the pediatric literature, all statements achieved high levels of consensus already after the first round and were confirmed after the second. This may reflect the awareness of unmet needs generated by COVID-19 lockdown periods. Indeed, some telemedicine programs that were already existing for chronic diseases, such as diabetes, hypothyroidism, inflammatory bowel diseases, celiac disease, cystic fibrosis, and epilepsy, have been further implemented during the pandemic. The issue of telemedicine was discussed in depth during the board meetings, and there was unanimous consensus that telemedicine might be useful for the management of chronic clinical diseases, monitoring of therapies, and giving advice to parents of children living in remote areas, therefore experiencing access difficulties to reach the hospital or their primary care pediatricians. However, it should be used carefully for acute diseases, such as infectious diseases, and for emergencies. In the case of febrile children, the board agreed that remote visits may help in collecting history before ambulatory visits, making a first diagnosis, and monitoring certain parameters. However, it must be kept in mind that the management cannot be disconnected from the ultimate diagnosis of the underlying disease, and one must be sufficiently skilled to quickly realize whether patient's conditions are deteriorating, thus requiring an urgent ambulatory or hospital visit with physical examination.

### Limitations

This study has some limitations. First, the questionnaire included only statements considering paracetamol and ibuprofen without multiple-choice questions giving panelists the opportunity of giving opinions on other antipyretic drugs, which may have induced bias. Second, the panel included a small number of pediatricians, all working in Italy. This may limit the generalizability of the results to all specialists within Italy or abroad. However, it should be noted that all the panel members had high clinical experience and their responses were most likely based on the most recent knowledge in the field.

Currently, there is no gold standard for sample size for Delphi panels. In published studies, the size can range from 10 to 1,000 participants (19), and many Delphi panels have been conducted on fewer than 15 participants (20).

Furthermore, according to experts, the success of a survey using the Delphi approach stems more from the qualification of the participating experts rather than the sample size (21).

### Conclusions

This consensus initiative using the Delphi process revealed a high level of consensus among Italian pediatricians on several aspects of the management of febrile children. In particular, there was consensus on the adequacy of all statements reflecting the recommendations given by regulatory agencies and guidelines, and on the position of paracetamol as first-line choice symptomatic treatment of fever in children. Moreover, there was consensus on the need of nationwide standardized implementation of telemedicine for management of selected conditions. Because of the limited number of participating pediatricians, the consensus statements of the present study should represent only expert opinions to support everyday decision process complementary to recommendations by regulatory agencies and existing guidelines.

## Data Availability Statement

The raw data supporting the conclusions of this article will be made available by the authors, without undue reservation.

## Author Contributions

All authors listed have made a substantial, direct, and intellectual contribution to the work and approved it for publication.

## Funding

This study received an unconditioned grant from Angelini Pharma S.p.A., Rome, Italy. The funder was not involved in the study design, collection, analysis, interpretation of data, and the writing of this article or the decision to submit it for publication.

## Conflict of Interest

The authors declare that the research was conducted in the absence of any commercial or financial relationships that could be construed as a potential conflict of interest.

## Publisher's Note

All claims expressed in this article are solely those of the authors and do not necessarily represent those of their affiliated organizations, or those of the publisher, the editors and the reviewers. Any product that may be evaluated in this article, or claim that may be made by its manufacturer, is not guaranteed or endorsed by the publisher.
