# Supplementary material for: The Effective Management of Fever in Pediatrics and Insights on Remote Management: Experts' Consensus Using a Delphi Approach
Source: Front Pediatr. 2022 Apr 26;10:834673. doi: 10.3389/fped.2022.834673 (PMC9087841; doi:10.3389/fped.2022.834673)
Supplement: Supplementary file 1 [file Data_Sheet_1.pdf]

Appendix

Coordinator
Vitale Antonio, Pediatric Unit AO San Giuseppe Moscati, Avellino.

Board (in alphabetical order)
Badolato Raffaele, Pediatric Clinic, University of Brescia and ASST-Spedali Civili of Brescia, Brescia.
Becherucci Paolo, Pediatric Primary Care, National Pediatric Health Care System, Lastra a Signa (Firenze).
Carreddu Domenico, Pediatric Primary Care, National Pediatric Health Care System, Novara.
Chiappini Elena, Pediatric Infectious Disease Unit, Anna Meyer Children's University Hospital, Firenze.
Di Mauro Antonio, Pediatric Primary Care, National Pediatric Health Care System, Margherita di Savoia (Barletta-Andria-Trani).
Doria Mattia, Pediatric Primary Care, National Pediatric Health Care System, Chioggia (Venezia).
Staiano Annamaria, Department of Translational Medical Science, Section of Pediatrics, University of Naples Federico II, Napoli.

Expert Panel those that partecipated at least 1 round Delphi - (in alphabetical order)

Alboresi Stefano, Pediatric Primary Care, National Pediatric Health Care System, Bologna.

Arcageli Bruno, Pediatric Primary Care, National Pediatric Health Care System, Pesaro Urbino.

Aloi Giuseppe, Pediatric Primary Care, National Pediatric Health Care System, Napoli.

Arrighi Antonello, Pediatric Primary Care, National Pediatric Health Care System, San Giovanni Val d'Arno (Arezzo).

Balzer Elena, Pediatric Primary Care, National Pediatric Health Care System, Lastra a Signa (Firenze).

Betta Marta, Pediatric Primary Care, National Pediatric Health Care System, Riva del Garda (Trento).

Bracaloni Davide, Pediatric Primary Care, National Pediatric Health Care System, Novara.

Brognoli Marika, Pediatric Unit, Pediaric Unit Policlinico Riuniti, Foggia.

Brusa Sandra, National Pediatric Health Care System, Imola.

Cacita Marilena, Pediatric Primary Care, National Pediatric Health Care System, Suno (Novara).

Calvi Donatella, Pediatric Primary Care, National Pediatric Health Care System, Torino.

Campanozzi Angelo, Pediaric Unit Policlinico Riuniti Foggia.

Cantelmi Grazia, Pediatric Primary Care, National Pediatric Health Care System, Napoli.

Cappiello Annarita, Pediatric Unit, San Paolo Hospital, Bari.

Carlomagno Francesco, Pediatric Primary Care, National Pediatric Health Care System, Napoli.

Carpino Antonio, Pediatric Primary Care, National Pediatric Health Care System, Napoli.

Castronuovo Serenella, Pediatric Primary Care, National Pediatric Health Care System, Roma.

Cazzato Teresa, Pediatric Primary Care, National Pediatric Health Care System, Taranto.

Corallo Paola Carmela, Pediatric Primary Care, National Pediatric Health Care System, Cisternino, Brindisi.

Cravidi Claudio, Pediatric Primary Care, National Pediatric Health Care System, Pavia.

Cresta Lorenzo, Pediatric Primary Care, National Pediatric Health Care System, Genova.

Crisci Valeria, Pediatric Unit A.O. San Giuseppe Moscati, Avellino.

D'Avino Antonio, Pediatric Primary Care, National Pediatric Health Care System, Napoli.

De Franchis Raffaella, Pediatric Primary Care, National Pediatric Health Care System, Napoli.

Dotta Laura, Pediatric Unit Ospedale dei Bambini ASST Spedali Civili, Brescia.

Farris Evelina, Pediatric Primary Care, National Pediatric Health Care System, Napoli.

Ferrara Dante, Pediatric Primary Care, National Pediatric Health Care System, Palermo.

Filippi Lorena, Pediatric Primary Care, National Pediatric Health Care System, San Michele all'Adige (Trento).

Filograna Mariarosaria, Pediatric Primary Care, National Pediatric Health Care System, Nardò (Lecce).

Frison Ezio, Pediatric Primary Care, National Pediatric Health Care System, Noale (Venezia).

Frongia Gianfranco, Pediatric Primary Care, National Pediatric Health Care System, Rignano sull'Arno (Firenze).

Furcolo Giuseppe, Pediatric Unit AO San Giuseppe Moscati, Avellino.

Gallo Patrizia, Pediatric Primary Care, National Pediatric Health Care System, Napoli.

Greco Luigi, Pediatric Primary Care, National Pediatric Health Care System, Bergamo.

Greco Silvia, Pediatric Department, G. D'Annunzio University, Chieti.

Gulino Antonio, Pediatric Primary Care, National Pediatric Health Care System, Misterbianco (Catania).

Iasevoli Salvatore, Pediatric Primary Care, National Pediatric Health Care System, Napoli.

Intini Silvia, Pediatric Primary Care, National Pediatric Health Care System, Bari.

La Marca Angela, Pediatric Unit AO San Giuseppe Moscati, Avellino.

Lamborghini Adima, Pediatric Primary Care, National Pediatric Health Care System, Teramo.

Landi Massimo, Pediatric Primary Care, National Pediatric Health Care System, Torino.

Leone Silvia, Pediatric Primary Care, National Pediatric Health Care System, Novara.

Libranti Maria, Pediatric Primary Care, National Pediatric Health Care System, Tremestieri Etneo (Catania).

Limongelli Antonio, Pediatric Primary Care, National Pediatric Health Care System, Atripalda (Avellino).

Maiorana Maria, Pediatric Unit AO San Giuseppe Moscati, Avellino.

Marconi Carlo, Pediatric Primary Care, National Pediatric Health Care System, Pesaro Urbino.

Marsella Maria, Pediatric Unit AO San Giuseppe Moscati, Avellino.

Matarazzo Maria, Pediatric Unit AO San Giuseppe Moscati, Avellino.

Mazzola Giuseppe, Pediatric Primary Care, National Pediatric Health Care System, Catania.

Mencacci Michele, Pediatric Primary Care, National Pediatric Health Care System, Magione (Perugia).

Minella Raffaele, Pediatric Primary Care, National Pediatric Health Care System, Napoli.

Monaci Alessandro, Pediatric Primary Care, Pediatric Unit Hospital Misericordia, Grosseto.

Naccari Anna, Pediatric Primary Care, National Pediatric Health Care System, Padova.

Napolitano Carlo, Pediatric Primary Care, National Pediatric Health Care System, Milano.

Osama Al Jamal, Pediatric Primary Care, National Pediatric Health Care System, Villasor (Cagliari).

Panza Raffaella, Pediatric Unit Azienda Consorziale Policlinico, Bari.

Pensabene Licia, Department of Surgical and Medical Sciences Magna Graecia University, Catanzaro.

Praitano Massimiliano, Pediatric Primary Care, National Pediatric Health Care System, Ostuni (Brindisi).

Pugliese Annalisa, Pediatric Unit AO San Giuseppe Moscati, Avellino.

Poli Piercarlo, Pediatric Unit Ospedale dei Bambini ASST Spedali Civili, Brescia.

Rispoli Giuliana, Pediatric Primary Care, National Pediatric Health Care System, Napoli.

Rizzo Valentina, Pediatric Unit AO Consorziale Policlinico, Bari.

Romano Roberta, Pediatric Primary Care, National Pediatric Health Care System, Napoli.

Russo Paola, Pediatric Unit AO San Giuseppe Moscati, Avellino.

Sannino Ciro, Pediatric Primary Care, National Pediatric Health Care System, Napoli.

Sartorello Francesca, Pediatric Primary Care, National Pediatric Health Care System, Novara.

Schiano di Cola Roberta, Università degli Studi della Campania Luigi Vanvitelli, Napoli.

Simeone Domenico, Pediatric Primary Care, National Pediatric Health Care System, Benevento
Timpano Silvana, Pediatric Unit Ospedale dei Bambini ASST Spedali Civili, Brescia.

Trapani Gianfranco, Pediatric Primary Care, National Pediatric Health Care System, Sanremo (Imperia).

Turco Rossella, A.O.R.N. Santobono Pausilipon - Ospedale Santobono Napoli

Turra Renato, Pediatric Primary Care, National Pediatric Health Care System, Caselle Torinese, Torino.

Urgesi Stefania, Pediatric Primary Care, National Pediatric Health Care System, Napoli.

Vesco Daniela Maria, Pediatric Primary Care, National Pediatric Health Care System, Castelletto sopra Ticino, Novara.

Zavallone Annalisa, Pediatric Primary Care, National Pediatric Health Care System, Tollegno (Biella).
